# Tomato Pomace Valorization: Green Solvent Evaluation, Microwave-Assisted Extraction Optimization, and Life-Cycle Assessment for Carotenoid Recovery

**DOI:** 10.3390/foods15203710

**Published:** 2026-10-18

**Authors:** Tea Petković, Nikolina Golub, Emerik Galić, Kristina Radić, Mario Jug, Nada Smigic, Ilija Djekić, Dubravka Vitali Čepo

**Affiliations:** 1 Department of Nutrition and Dietetics, University of Zagreb Faculty of Pharmacy and Biochemistry, Ante Kovačića 1, 10000 Zagreb, Croatia; tea.petkovic@pharma.unizg.hr (T.P.); nikolina.golub@pharma.unizg.hr (N.G.); emerik.galic@pharma.unizg.hr (E.G.); kristina.radic@pharma.unizg.hr (K.R.); 2 Department of Pharmaceutical Technology, University of Zagreb Faculty of Pharmacy and Biochemistry, Ante Kovačića 1, 10000 Zagreb, Croatia; mario.jug@pharma.unizg.hr; 3 Department of Food Safety and Quality Management, University of Belgrade Faculty of Agriculture, Nemanjina Street 6, 11080 Belgrade, Serbia; nadasmigic@agrif.bg.ac.rs (N.S.); idjekic@agrif.bg.ac.rs (I.D.)

**Keywords:** tomato pomace, eutectic solvent, green extraction, microwave-assisted extraction, β-carotene, lycopene

## Abstract

This study investigated the recovery of β-carotene (BC) and lycopene (LYC) from tomato processing waste (TPW) using microwave-assisted extraction (MAE) with green solvents. Sunflower oil (SO), olive oil, limonene, and several deep eutectic solvents were initially screened, after which SO and a capric acid–lauric acid deep eutectic solvent (CLA; 2:1 molar ratio) were selected for further optimization. The optimized MAE conditions for SO were 217 W for 4 min, yielding 2.82 ± 0.24 mg/100 g dry TPW of BC and 3.57 ± 0.27 mg/100 g of LYC, with an energy consumption of 0.014 kWh. For CLA, the selected conditions were 1800 W for 13 min, which resulted in significantly higher recoveries of BC (5.29 ± 0.84 mg/100 g) and LYC (9.80 ± 1.77 mg/100 g), although the energy consumption was higher (0.390 kWh). Vibrational micro-milling reduced the median particle size from approximately 303 to 66.8 μm and improved carotenoid recovery when combined with SO; however, it decreased recovery in CLA under more intensive MAE conditions. Life-cycle assessment (LCA) comparing optimal MAE extraction to conventional solvent extraction and ultrasound-assisted extraction showed that the CLA-based MAE process provided the most favorable overall balance between carotenoid recovery and environmental performance. Therefore, CLA can be recommended as the most effective solvent for the MAE recovery of BC and LYC from TPW, although further validation at a pilot scale and an assessment of solvent recovery are required.

## 1. Introduction

The rapid expansion of tomato production and processing has resulted in the generation of considerable quantities of tomato processing waste (TPW), typically including tomato skins, seeds, vascular tissues, and residual flesh. Depending on the processing technology and the characteristics of the final product, these residues may represent approximately 2–10% of the initial tomato mass, generating several million tonnes of TPW annually [1,2]. Its disposal remains associated with substantial management costs and may contribute to environmental pollution, greenhouse gas emissions, and the inefficient use of agricultural resources [1]. From a circular economy perspective, recovering valuable constituents from TPW could therefore reduce the environmental burden of waste while creating new sources of industrial raw materials.

At present, TPW is used primarily as animal feed, soil amendment, fertilizer, or a substrate for energy-related applications. However, in recent years, tomato residues have been of particular interest due to their high content of carotenoids, which have been investigated in relation to numerous health-promoting effects and can be incorporated into functional foods, pharmaceutical products, and cosmetic formulations [3]. This approach represents a valuable strategy consistent with major principles of the circular economy that prioritize waste prevention, reuse, recycling, and the recovery of valuable materials over disposal [4].

Accordingly, further research should focus on the development of environmentally preferable extraction processes based on novel solvents, such as deep eutectic solvents (DESs), bio-based solvents, and supercritical fluids, and innovative techniques, including ultrasound-assisted extraction (UAE), microwave-assisted extraction (MAE), pulsed electric field extraction (PEF), and pressurized liquid extraction (PLE) [5,6,7].

Recent literature data show that green extractions using GRAS (generally recognized as safe) solvents can be an effective alternative to improve extraction procedures and optimize carotenoid extraction, emphasizing the use of SCFs (short-chain fatty acids), limonene, vegetable oils, and DESs [8,9,10,11,12,13,14]. Particular interest has lately been paid to the application of DESs, mostly due to their high accessibility, simple preparation, low toxicity, biodegradability, and the fact that their physicochemical properties can be tailored by modifying the constituent compounds and adjusting their molar ratios [8]. Hydrophobic DESs are especially suitable for lycopene and other nonpolar carotenoids, while their hydrogen-bonding capacity may promote matrix disruption, solubilization, and protection of the extracted pigments against oxidation. DES-based extraction protocols for carotenoids from different types of tomato waste are listed in Table 1.

Comparative studies have reported that selected hydrophobic DESs can provide carotenoid recoveries from different types of raw materials comparable to or higher than those obtained using hexane and vegetable oils, although extraction performance is strongly dependent on the solvent composition and operating conditions [25,26,27]. Nevertheless, DESs present several limitations that may restrict their practical application. Their high viscosity can reduce solvent penetration, impair mixing, and limit mass transfer, while the addition of water to decrease viscosity may alter the eutectic structure and reduce the extraction capacity for hydrophobic carotenoids. Furthermore, their negligible volatility makes solvent removal and carotenoid purification more difficult than in conventional solvent systems, potentially increasing downstream processing costs.

According to a recent review, most currently available green extraction protocols for carotenoid extraction rely either on supercritical fluid extraction (SFE (37%) or UAE (30%), with UAE predominantly used for tomatoes and tomato industry byproducts [26]. Compared to UAE, MAE may provide more rapid and homogeneous heating and is relatively simple to integrate with edible oils, ethanol–water systems, or other green solvents. In comparison to many other green extraction techniques, MAE usually requires lower capital investment and less complex high-pressure equipment, while offering easier adaptation to different solid matrices. Importantly, MAE has progressed beyond the laboratory proof-of-concept stage and shows substantial potential for semi-industrial and industrial application, particularly through continuous-flow or modular reactor designs [27].

To our knowledge, the suitability of MAE for the green extraction of carotenoids from TPW has not been thoroughly investigated. Several authors [12,13,14] used MAE with ethyl acetate (or its mixtures with ethanol) to obtain extracts rich in β-carotene and lycopene. In those studies, MAE generally provided higher carotenoid yields in significantly shorter time than CSE or other techniques, such as high-pressure-assisted extraction (HPAE), indicating its potential as an alternative to conventional extraction. Also, the applicability of other green solvents for the MAE extraction of carotenoids from TPW has not been investigated so far.

Despite promising results and greater efficiency than CSE or HPAE, MAE’s environmental performance is not inherently sustainable. The overall impact may be influenced by the electricity demand of microwave generation, cooling, stirring, centrifugation, and solvent recovery, as well as by the solvent selected, equipment manufacture, extraction yield, scale-up efficiency, and the electricity mix. Therefore, the environmental impact of an MAE procedure should be quantified using an objective, standardized methodology such as a life-cycle assessment (LCA), with a clearly defined functional unit, system boundaries, life-cycle inventory, and multiple impact categories, rather than being inferred solely from reduced extraction time or solvent use [28,29].

Vibrational micro-milling has been proven as a promising mechanical pretreatment for improving carotenoid recovery by reducing particle size, increasing the specific surface area, and disrupting the plant matrix, thereby facilitating solvent penetration and mass transfer during subsequent green extraction [1,26,30]. Since its effect depends on the initial moisture content, milling intensity, treatment duration, and protection from heat, light, and oxygen, which are important because excessive mechanical treatment may promote carotenoid degradation, its potential should be systematically evaluated and optimized specifically for carotenoid recovery from TPW rather than inferred from studies targeting other compounds [1,26,30]. In the case of TPW freeze-drying followed by milling, enzymatic hydrolysis, pulsed-electric-field treatment, and ultrasonication have been reported to enhance lycopene and total carotenoid yields [31,32].

This paper aims to provide insights into the comparative effectiveness of different green solvents (limonene, DESs, vegetable oils) for the MAE of TPW-derived carotenoids, provide optimal extraction conditions for β carotene and lycopene (considering their chemical differences), and investigate the potential of TPW pretreatment for improving carotenoid yields. Moreover, it compares MAE with UAE and CSE, considering yields but also the ecological footprint as calculated by an LCA. The obtained results enable the selection of processes that are not only efficient but also environmentally preferable across the full value chain. Collectively, the paper provides a rigorously evaluated, scalable MAE process and clarifies its presumed comparative advantages compared to other extraction methods. It validates vibrational micro-milling as an effective pretreatment for TPW and provides an LCA-backed sustainability profile of the carotenoid extraction process, thereby advancing both the science and practical implementation of circular economy strategies for tomato byproduct valorization.

## 2. Materials and Methods

### 2.1. Organization of Research

**Figure 1:**
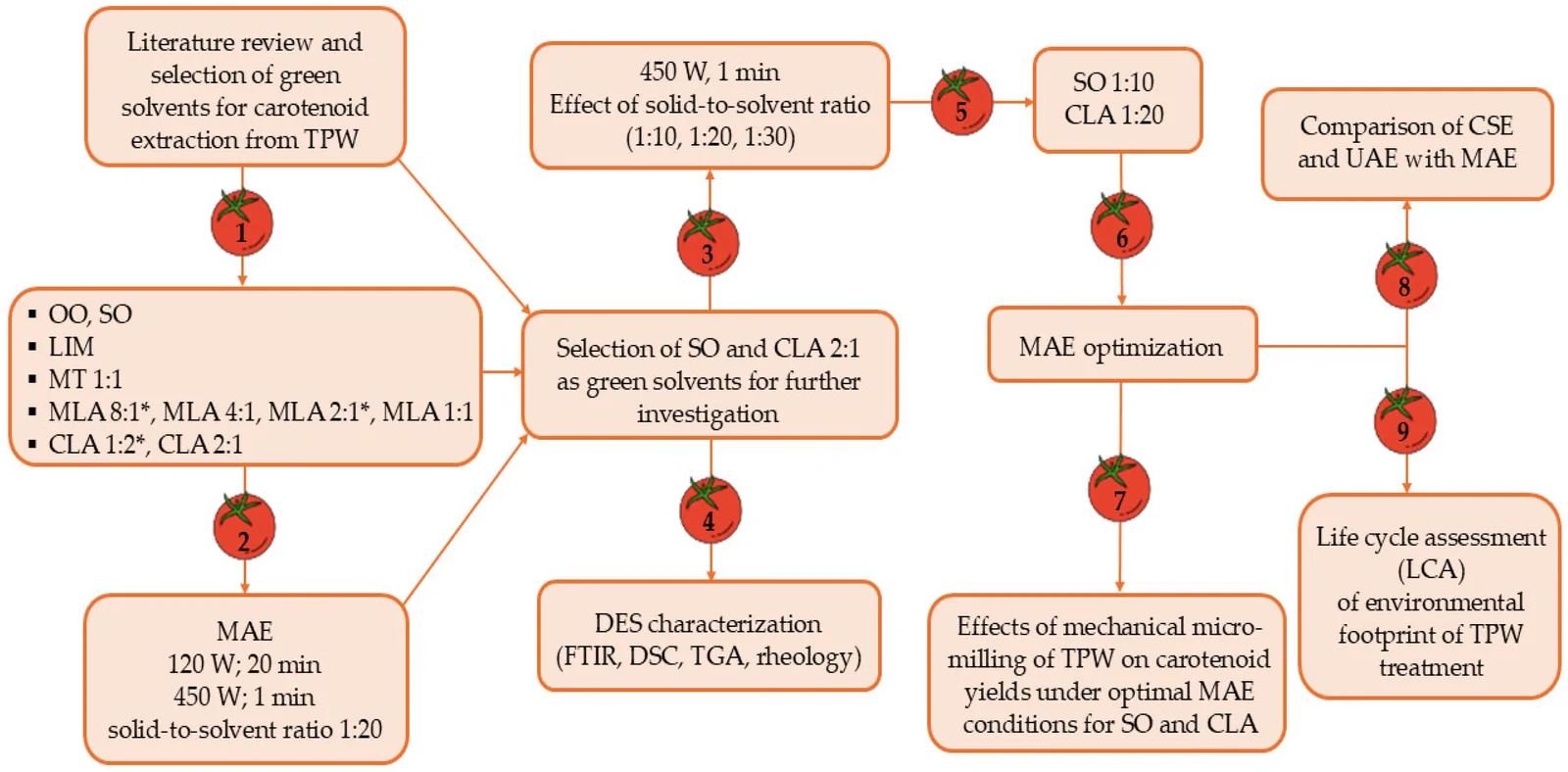
Flowchart of the experimental workflow. TPW: tomato processing waste; OO: olive oil; SO: sunflower oil; LIM: limonene; MT: menthol and thymol; MLA: menthol and lactic acid; CLA: capric and lauric acids; MAE: microwave-assisted extraction; DES: deep eutectic solvent; FT-IR: Fourier-transform infrared spectroscopy; DSC: differential scanning calorimetry; TGA: thermogravimetric analysis; CSE: conventional solvent extraction; UAE: ultrasound-assisted extraction; LCA: life-cycle assessment. Solvents marked with an asterisk were not selected for further research due to unsuccessful DES formation and instability at room temperature.

This research was conducted in several stages, as shown in Figure 1.

Based on a comprehensive literature search, the following GRAS solvents were selected for the preliminary phase of the research: olive oil (OO), sunflower oil (SO), limonene (LIM) and deep eutectic solvents (DESs) prepared at several molar ratios (menthol and thymol [MT] at 1:1; menthol and lactic acid [MLA] at 8:1, 4:1, 2:1, and 1:1; and capric and lauric acids [CLA] at 1:2 and 2:1). Their efficiency was tested using MAE under two sets of chosen conditions, based on available literature data (120 W, 20 min; 450 W, 1 min). Extraction solvents providing the highest carotenoid yields were chosen for further MAE optimization. A literature-guided, sequential exploration of MAE extraction was conducted separately for SO and CLA. Extraction time and microwave power were selected as the principal process variables, while lycopene and β-carotene yields as well as energy consumption were used as response criteria. The initial experimental ranges were selected from published extraction conditions because directly transferable data for the MAE of tomato processing waste using the investigated solvents were unavailable. The selected conditions therefore represent the best-performing experimentally evaluated conditions within the investigated operating window, rather than a statistically model-derived global optimum.

The possibilities for further improvement of extraction yields through particle size reduction by vibrational micro-milling were investigated. Optimized MAE procedures for both extraction solvents were compared to CSE and UAE in terms of yields and environmental footprint (investigated using an LCA approach). For UAE, the conditions were previously described in the literature by other authors, and hexane–acetone CSE was chosen as the conventional reference extraction for recovering carotenoids and other lipophilic compounds.

### 2.2. Chemicals

The olive oil (OO) used for the preliminary research was supplied by a small local producer, and sunflower oil from the Croatian edible oil manufacturing and processing company Zvijezda (Zagreb, Hrvatska) was purchased in a store. For the preparation of the DESs, decanoic (capric) acid (98%; BLDpharm, Shanghai, China), dodecanoic (lauric) acid (99%; Thermo Fisher Scientific, Waltham, MA, USA), L-menthol Ph. Eur. (T. T. T., Sveta Nedelja, Croatia), thymol p.a. (T. T. T., Sveta Nedelja, Croatia), and 90% lactic acid in aqueous solution Ph. Eur. (VWR Chemicals, Radnor, PA, USA) were used. Potassium hydroxide was used for saponification. Methyl tert-butyl ether for HPLC (MTBE) (VWR Chemicals, Radnor, PA, USA), tetrahydrofuran (THF; ≥99.0% with 250 ppm BHT as an inhibitor; VWR Chemicals, Radnor, PA, USA), and n-hexane reag. Ph. Eur. (Carlo Erba, Milan, Italy) were used for liquid–liquid partitioning, phase separation, and solvent exchange, while 2-propanol for HPLC (VWR Chemicals, Radnor, PA, USA) was used to reconstitute the evaporated extract prior to HPLC analysis. The previously mentioned n-hexane Ph. Eur. and acetone p. a. (GRAM MOL, Zagreb, Croatia) were used for conventional solvent extraction (CSE). Ammonium acetate reag. Ph. Eur. (Supelco, Bellefonte, PA, USA), methanol for HPLC (≥99.9%; Honeywell, Charlotte, NC, USA), and the previously mentioned MTBE were used for the preparation of the mobile phases for HPLC analysis. Standards of β-carotene (TCI, Tokyo, Japan) and lycopene (Extrasynthese, Lyon, France) were used for the detection of carotenoids and the preparation of calibration curves.

### 2.3. Tomato Processing Waste (TPW)

The TPW was obtained after the heat treatment (boiling for 60 min) and juice separation of ripe tomatoes collected from the experimental fields of the Faculty of Agrobiotechnical Sciences Osijek, J. J. Strossmayer University of Osijek, Croatia. It was stored in plastic bags at −4 °C until further use. Prior to drying, the TPW was thawed, divided into smaller portions, and weighed. The material was then spread in a thin layer onto baking paper using a spatula to facilitate faster and more uniform drying. Based on previous preliminary experiments [33], the TPW was dried at 70 °C in a laboratory dryer (Inko, Zagreb, Croatia) on two shelves with controlled airflow for 14.5 h. After drying, the material was manually crushed, weighed, ground using a conventional mill (Bosch, Munich, Germany) for 18 s, and stored at +4 °C for subsequent use. The TPW from two vegetation years was used: 2023/2024 (for preliminary experiments) and 2024/2025 (for MAE optimization and UAE/CSE/MAE comparison).

### 2.4. Preparation and Characterization of Deep Eutectic Solvents

#### 2.4.1. Preparation of DESs

Preparation of the DESs was conducted as presented in Table 2 based on procedures described by different authors [16,18,19]. The prepared DESs were transferred to sealed glass vials and stored at room temperature until the experiment. At room temperatures below 17 °C, the DESs composed of menthol–lactic acid and capric acid–lauric acid solidified or partially solidified during storage. Before use, the systems were remelted in a water bath maintained at 25–30 °C. The MT mixture was stable at temperatures both below and above room temperature. To prevent solidification during the experiments, the CLA mixture was maintained at 51 °C under continuous magnetic stirring at 200 rpm.

#### 2.4.2. Characterization of DES

##### Fourier-Transform Infrared (FT-IR) Spectroscopy

The FT-IR spectrum of the DESs was recorded using a Nicolet Summit X spectrometer (Thermo Fisher Scientific, Waltham, MA, USA) equipped with an Everest ATR (attenuated total reflectance) accessory. Spectra were collected over the range 4000–400 cm^−1^ at a resolution of 4 cm^−1^, with 32 scans per sample, including 16 background scans. Prior to each sample measurement, background scans were collected to minimize the influence of environmental interference and instrument noise. The spectra were analyzed using OMNIC Paradigm software (version 2.6.1057).

##### Differential Scanning Calorimetry (DSC)

The thermal properties of the DESs were determined using a Discovery DSC 250 Differential Scanning Calorimeter (TA Instruments, New Castle, DE, USA). DSC was used to determine the melting point, crystallization behavior, and heat flow changes by monitoring the difference in heat flow between the sample and a reference material during controlled heating and cooling. An empty aluminum pan with a pierced lid was used as the reference. Approximately 2–10 mg of sample (capric acid, lauric acid, physical mixture, and eutectic solvent) was weighed into an aluminum pan, hermetically sealed with a pierced aluminum lid, and placed into the oven of the DSC instrument under a nitrogen flow of 50 mL/min. The following analysis procedure was applied: equilibration at 0 °C, followed by an isothermal step for 5 min and a heating ramp of 5 °C per minute up to 50 °C. Normalized heat flow (W/g) was recorded as a function of temperature, and the thermograms obtained were analyzed using TRIOS^TM^ software (version 5.1.1.46572) (TA Instruments, New Castle, DE, USA).

##### Thermogravimetric Analysis (TGA)

The thermal stability of the DESs under controlled heating conditions was evaluated using a Discovery TGA 550 thermogravimeter (TA Instruments, New Castle, DE, USA). The analysis was performed at a heating rate of 10 °C/min up to 500 °C. Approximately 10 mg of the analyzed sample was placed into a platinum pan placed on a high-precision balance and then inserted into the TGA instrument oven. The results are presented as TGA and DTG curves and were analyzed using TRIOS^TM^ software (version 5.1.1.46572) (TA Instruments, New Castle, DE, USA).

##### Determination of Rheological Properties

The rheological properties of the DESs were determined using a Modular Compact Rheometer MCR 102 (Anton Paar GmbH, Graz, Austria) equipped with a 50 mm cone-plate geometry (CP50, cone angle 1°). Flow curves were measured in triplicate at 25 °C over the selected shear-rate range (0.1–1000 s^−1^). The dependence of viscosity on shear rate was evaluated, and the zero-shear viscosity was estimated using the Carreau–Yasuda model. Data analysis was performed using RheoCompass™ Light software (version 1.23.403) (Anton Paar GmbH, Graz, Austria).

### 2.5. Carotenoid Extraction

#### 2.5.1. Conventional Solvent Extraction (CSE)

CSE was performed according to the procedure described by Kyriakoudi and co-authors [16]. Extraction was performed in glass vials, using an IKA RT 15 magnetic stirrer (IKA, Staufen, Germany) at 550 rpm for 30 min at room temperature in the dark. Several solvents were utilized (hexane–acetone (HA) 1:1 (*v*/*v*), SO, and CLA) using a solid-to-solvent ratio of 1:20 (0.5 g in 10 mL). The extracts were transferred to 50 mL Falcon tubes and centrifuged at 4400 rpm for 10 min. Saponification, liquid–liquid partitioning, phase separation, and solvent exchange were performed according to the method described by Hostetler [34], with slight modifications. Experiments were performed in triplicate (*n* = 3). All results are expressed in mg per 100 g of dry TPW.

#### 2.5.2. Ultrasound-Assisted Extraction (UAE)

UAE was performed according to a previously optimized procedure described by Marianccio and co-authors [18]. Extraction was performed in a Sonorex Digiplus DL 510 H ultrasonic bath (Bandelin, Berlin, Germany) for 30 min at 50 °C and 100% ultrasound power (35 kHz/640 W) in the dark using SO and CLA as the extraction solvents. The extraction was carried out using a solid-to-solvent ratio of 1:20 (0.5 g in 10 mL) in 100 mL glass beakers. Experiments were performed in triplicate (*n* = 3). The extracts underwent the same post-extraction treatment as described for CSE.

#### 2.5.3. Microwave-Assisted Extraction (MAE)

MAE was performed using the Ethos Easy MLS1200 Mega microwave system (Milestone, Sorisole, Italy). TPW samples in SO were prepared in Teflon vessels at a solid-to-solvent ratio of 1:10 (0.5 g TPW in 5 mL SO), whereas samples in CLA were prepared at a ratio of 1:20 (0.25 g TPW in 5 mL DES). The vessels were then sealed and placed in the microwave oven. Carotenoid extractions were carried out under several combinations of extraction time and microwave power, ranging from 1 to 13 min and from 100 to 1800 W, respectively, depending on the extraction solvent, as shown in Table 3. All extractions began with a 10 s ramp to reach the defined power, followed by a constant-power phase for the designated extraction time, and ended with a 5 min cooling phase. At more extreme microwave power (1600 W and 1800 W), extractions began with 20 s to reach the defined power and ended with a 10–25 min cooling phase. Extractions were conducted in triplicate (*n* = 3). The extracts underwent the same post-extraction procedure as described for CSE.

##### Optimization of MAE

The optimization for SO and CLA included time (min) and microwave power (W) as the input variables, while the response variables were lycopene (LYC) and β-carotene (BC) yields (mg/100 g of dry mass), as well as energy expenditure. The initial time/power range was determined based on a literature review. However, in the case of CLA, after the completion of a set of reactions, the list was modified to include more extreme boundary conditions to identify the stability limits of the targeted compounds. The extractions were performed in triplicate, and the parameter values are presented in Table 3. Optimal conditions were selected based on the obtained yields and energy consumption. For CSE and UAE, optimal parameters reported in the literature were adopted without further modification.

### 2.6. Milling, Vibrational Micro-Milling, and Particle Size Analysis

Dried TPW was ground using a conventional mill for 18 s, as previously described [30], and a portion of the TPW was additionally treated using a vibrating MM 500 control micro-mill (Retsch, Haan, Germany). For this purpose, 8 g of TPW was placed into a 50 mL zirconium oxide grinding chamber with 24 small balls (5 mm) and 12 large balls (10 mm) and milled at 30 Hz for 15 min [30]. The milled TPW was stored at 4 °C.

The particle size distribution, expressed as a volume distribution, of the differently milled samples was determined using laser diffraction (Mastersizer 3000, Malvern Instruments, Worcestershire, UK). The instrument was equipped with a Hydro SV dispersion unit and a magnetic stirrer. A small amount of sample was dispersed in 10 mL of distilled water and added to the Hydro SV unit until 10–20% obscuration. The results are presented as an average of five replicates.

### 2.7. Chromatographic Determination of β-Carotene and Lycopene

Carotenoid separation and quantification were performed using reverse-phase high-performance liquid chromatography (RP-HPLC) on an Agilent 1260 Infinity II system (Agilent Technologies, Santa Clara, CA, USA) equipped with a diode array detector (DAD) according to the method described by Hostetler [34], with minor modifications. Chromatographic separation was achieved using a C30 Develosil column (5 µm particle size, 4.6 mm × 250 mm) (Phenomenex, Torrance, CA, USA). The flow rate was set at 1 mL/min, the column temperature was set at 30 °C, and the injection volume was set at 30 μL. The mobile phase system consisted of two solvents: (A) 20 mM ammonium acetate in methanol– water (98:2, *v*/*v*) and (B) methyl tert-butyl ether (MTBE). Gradient elution was performed as follows: 97% A to 92% A (0–1 min), 92% A to 85% A (1–8 min), 85% A to 0% A (8–25 min), held at 0% A for 0.5 min (25–25.5 min), and return to initial conditions (97% A) over 0.5 min (25.5–26 min). Spectral data were collected over the wavelength range of 190–800 nm, and carotenoids were detected at 450 and 470 nm. BC and LYC standards, together with their calibration curves, were used for quantification. The calibration curves exhibited a linear response over the concentration ranges of 0.005–0.08 µg per injection for β-carotene (y = 13,809.599x − 7229) and 0.00092–0.0736 µg per injection for lycopene (y = 19,222.554x − 4.356), with coefficients of determination (R^2^) of 0.9999 for both analytes (y = peak area, x = injected amount, µg). Limits of detection (LOD) and quantification (LOQ) were calculated from the calibration data using the criteria 3.3σ/S and 10σ/S, respectively. The resulting LOD and LOQ values were 0.0011 and 0.0034 µg per injection for β-carotene and 0.0013 and 0.0040 µg per injection for lycopene. Considering a sample injection volume of 30 µL, these values corresponded to concentrations of 0.037 and 0.11 µg/mL for β-carotene and 0.044 and 0.13 µg/mL for lycopene. Injection precision (*n* = 3), expressed as relative standard deviation (RSD), ranged from 0.11 to 1.44% for β-carotene and 0.02 to 0.39% for lycopene. Prior to analysis, all extracts were filtered through 0.22 μm PTFE membrane filters, and mobile phases were filtered through 0.45 μm cellulose membrane filters and degassed by ultrasonication for 15 min.

### 2.8. Environmental Assessment of Tomato Pomace Treatment Using Life-Cycle Assessment (LCA)

To assess the environmental footprint of the different extraction methods of carotenoids from tomato pomace, a partial life-cycle assessment (LCA) was performed using the “gate-to-gate” approach, in line with ref. [30] and according to LCA standards [35,36]. Three extraction methods were evaluated—conventional solvent extraction (CSE), microwave-assisted extraction (MAE), and ultrasound-assisted extraction (UAE)—in three different extraction solvents: SO (for all three extractions), CLA 2:1 (for all three extractions), and HA 1:1 (CSE). To perform this assessment, the main functional unit (FU) was defined as one extraction treatment of tomato pomace to enable comparison between the seven extraction treatments.

Two main inventories were used as inputs—the electric energy needed for sample preparation and treatment, and the chemicals used as solvents. Since the same equipment was used for drying the tomato pomace, mass allocation was applied to specify energy consumption. The output included dry tomato waste as solid organic waste. The environmental impact of tomato pomace (as input) was not included due to the small amounts used and to avoid the potential influence of this impact on the extraction process [37].

Environmental impacts were calculated using the ReCiPe hierarchist midpoint method, consistent with previous LCA studies [38,39]. Nine environmental impact indicators were selected to represent five main perspectives aligned with food-related LCA studies [38,40]: (i) climate change, comprising Global Warming Potential (GWP) and Ozone Depletion Potential (ODP); (ii) acidification, represented by Terrestrial Acidification (TAC); (iii) eutrophication, comprising Freshwater Eutrophication (FEP) and and Marine Eutrophication (MEP); (iv) toxicity, represented by Overall Toxicity (OTX), calculated as the sum of Human Toxicity (HTX), Freshwater Ecotoxicity (FTX), Marine Ecotoxicity (MTX), and Terrestrial Ecotoxicity (TTX); and (v) resource depletion, comprising Water Depletion (WDP), Fossil Fuel Depletion (FDP), and Agricultural Land Occupation (ALO). Calculation of the environmental footprint was performed using data from LCA databases (Environmental Footprint and Ecoinvent) available in openLCA (2024) (GreenDelta GmbH, Berlin, Germany).

Since the yield in each treatment was expressed in total carotenoids (mg/100 g of dry sample), the environmental footprints were re-calculated per 1 mg of total carotenoids. To compare the treatments, average values of the seven treatments were calculated to serve as reference points.

### 2.9. Data Analysis

Experiments were performed in duplicate, triplicate, or in six replicates, depending on the applied method of analysis. Statistical analysis of all data was performed using Microsoft Excel (Microsoft, Redmond, WA, USA) and GraphPad Prism 10.4.1 (Boston, MA, USA). The obtained results are presented as mean values ± standard deviation. Comparisons between the tested groups were performed using Student’s *t*-test or one-way ANOVA followed by Tukey’s post hoc multiple comparisons test, depending on the number of groups. Statistical significance was considered at *p* < 0.05. For the MAE optimization process, the Stat-Ease 360 program (version 25.0.5) (Minneapolis, MN, USA) was used.

## 3. Results and Discussion

### 3.1. Preliminary Investigation: Selection of Green Solvent and Determination of Solid-to-Solvent Ratio

#### 3.1.1. Selection of the Extraction Solvent

Recent comprehensive reviews indicate that green solvent extractions of carotenoids from tomatoes and tomato processing residues have primarily employed ethanol, ethyl lactate, ethyl acetate, and their mixtures, vegetable oils, limonene, and natural or hydrophobic DESs, including menthol–lactic acid and choline chloride- or fatty-acid-based systems. The extraction efficiency of these solvents depends on the extraction technique, operating conditions, and physicochemical properties of the target carotenoids (Table 1) [26,41].

**Figure 2:**
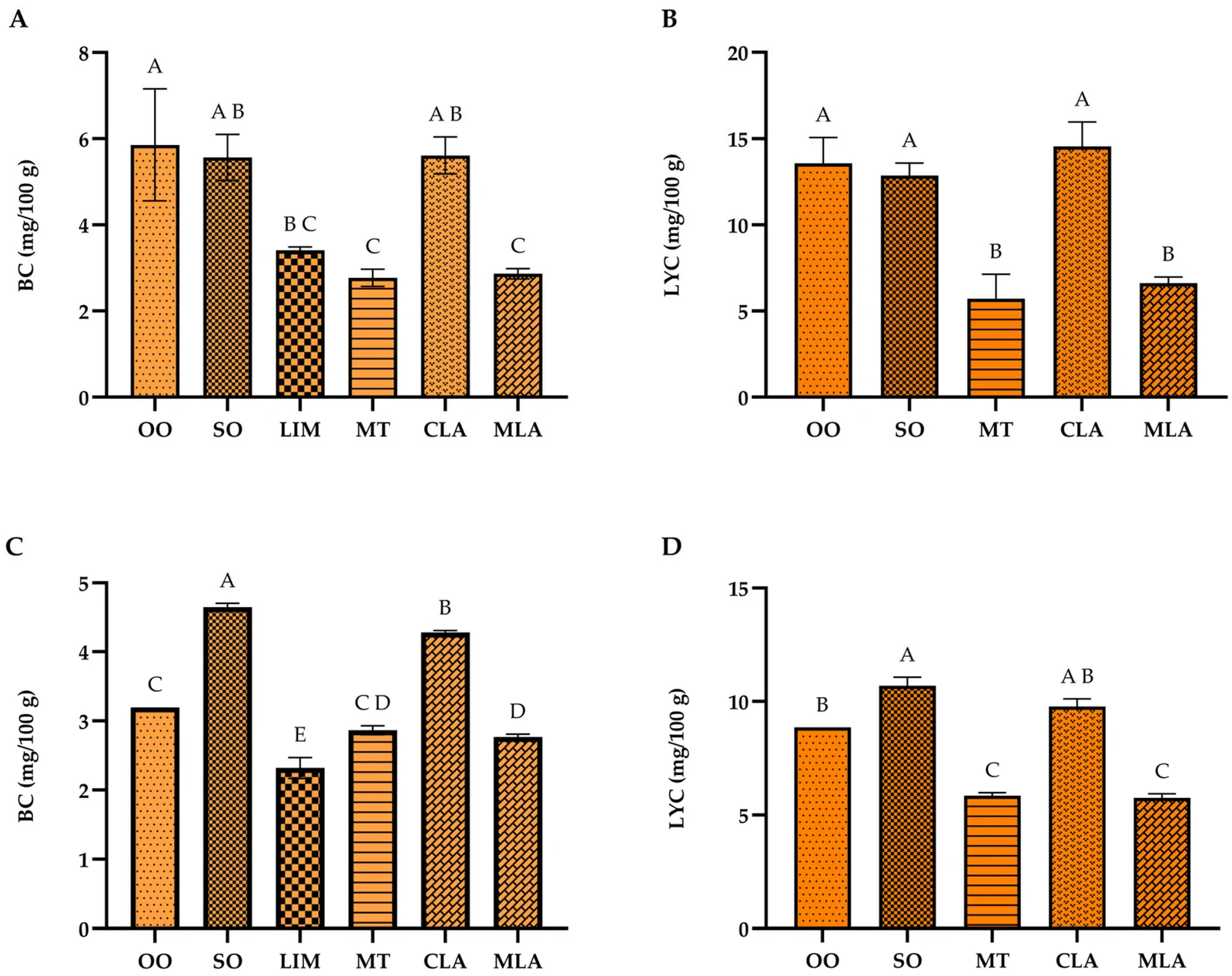
Comparative effectiveness of different green solvents for extraction of carotenoids from TPW depending on extraction conditions: (**A**) MAE 120 W, 20 min, BC; (**B**) MAE 120 W, 20 min, LYC; (**C**) MAE 450 W, 1 min, BC; (**D**) MAE 450 W, 1 min, LYC. Results are shown as mean ± SD. Statistical significance was evaluated using one-way ANOVA with Tukey’s post hoc test. Different letters above the bars indicate significant differences (*p* < 0.05) within each measured component. MAE: microwave-assisted extraction; OO: olive oil; SO: sunflower oil; LIM: limonene; MT: menthol–thymol 1:1; CLA: capric acid–lauric acid 2:1; MLA: menthol–lactic acid 4:1.

Since only ethyl acetate has been evaluated for the MAE of carotenoids from TPW so far, we investigated BC and LYC recovery using other green solvents selected based on their efficiency reported in the literature: OO, SO, LIM, MT (1:1), CLA (2:1), and MLA (4:1). Given that the suitability of a particular solvent may depend on the extraction conditions, the selected solvents were compared using MAE under low-power/long-time conditions (120 W for 20 min) and MAE under high-power/short-time conditions (450 W for 1 min) (Figure 2).

The highest BC yields were obtained under low-power/long-time MAE using SO, CLA, and OO (5.86 ± 1.30, 5.61 ± 0.63, and 5.56 ± 0.54 mg/100 g, respectively), with no significant differences among the tested solvents (*p* > 0.05). The same trend was observed for LYC, where the obtained yields ranged from 13.56 ± 1.49 mg/100 g in OO up to 14.54 ± 1.43 mg/100 g in CLA, with no significant differences in the efficiency of OO, SO, or CLA. Under low-power/long-time MAE, both tested vegetable oils provided comparable BC/LYC yields, while under high-power/short-time MO conditions SO was superior to OO.

Limonene was found to be the least suitable among the tested extraction solvents—limonene extracts did not contain LYC and provided one of the lowest yields of BC, particularly under high-power/short-time MAE conditions (2.32 ± 0.15 mg/100 g). The higher suitability of limonene for BC extraction compared to LYC extraction may be related to differences in molecular conformation, crystal packing, matrix accessibility, and stability during MAE. Although both carotenoids are highly lipophilic and can theoretically dissolve in limonene, the compact cyclic structure of BC may provide more favorable dispersion interactions with the nonpolar terpene solvent than the extended, linear polyene structure of LYC. Reported Hansen solubility parameter data indicate a closer solvent–solute affinity between limonene and BC, and BC solubility in limonene has been reported to be approximately 1.5-fold higher than in hexane [41]. LYC may be more difficult to extract because its linear, highly conjugated structure favors crystal packing and strong association with chromoplast and lipid structures. In addition, LYC may be more susceptible to thermal or oxidative degradation and isomerization during microwave treatment [42].

As pointed out previously, CLA provided the highest yields of both carotenoids among the tested DESs, regardless of the applied MAE conditions, and therefore was used for further MAE optimization.

**Figure 3:**
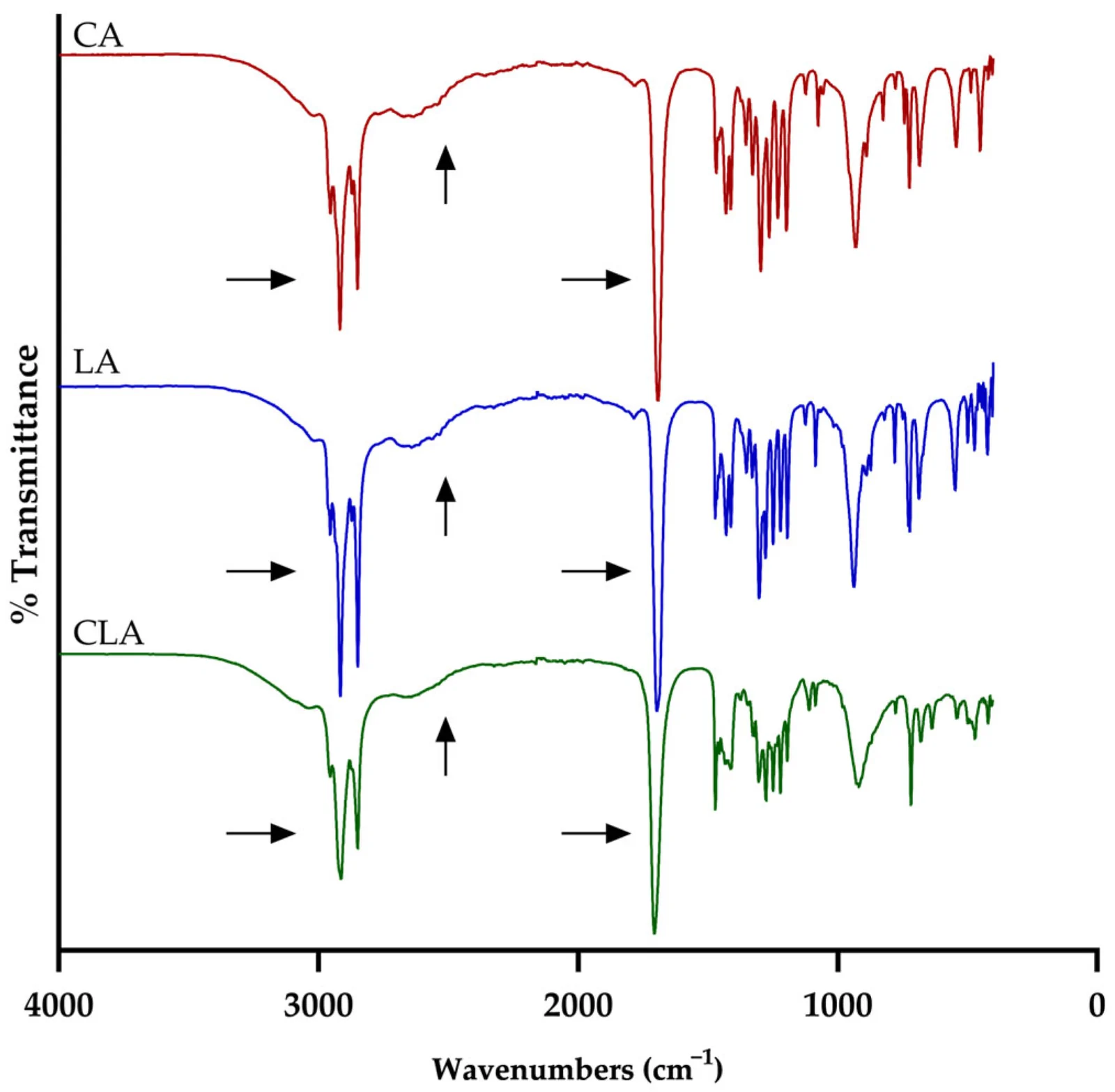
FT-IR spectra of pure capric acid (red spectrum), lauric acid (blue spectrum), and the prepared DES, CLA (green spectrum). CA: capric acid; LA: lauric acid; CLA: capric acid–lauric acid 2:1. Arrows indicate changes in the spectrum of the DES relative to the spectra of the pure components (CA and LA).

As presented in Figure 3, the spectroscopic evidence is consistent with the assumption that CLA is indeed an interacting eutectic system formed from CA and LA rather than a simple physical mixture. The FT-IR spectra of CA, LA, and their 2:1 mixture (CLA) showed the characteristic absorption bands of the individual fatty acids, including the broad O–H stretching region, C–H stretching bands, the carbonyl stretching band, and signals in the fingerprint region. In the FT-IR spectrum of CLA, these bands were retained but showed changes in band shape, relative intensity, and, for some signals, position compared to the pure components. These spectral differences indicate that the vibrational environment of the functional groups in CLA differs from that of the individual acids and are consistent with interactions between components. Such band broadening and wavenumber changes arise from the redistribution of electron density and the formation of an extended hydrogen-bond network between the two organic acids, which is consistent with the occurrence of molecular interactions during DES formation. However, FT-IR analysis alone does not allow the nature or strength of these interactions to be determined conclusively.

**Figure 4:**
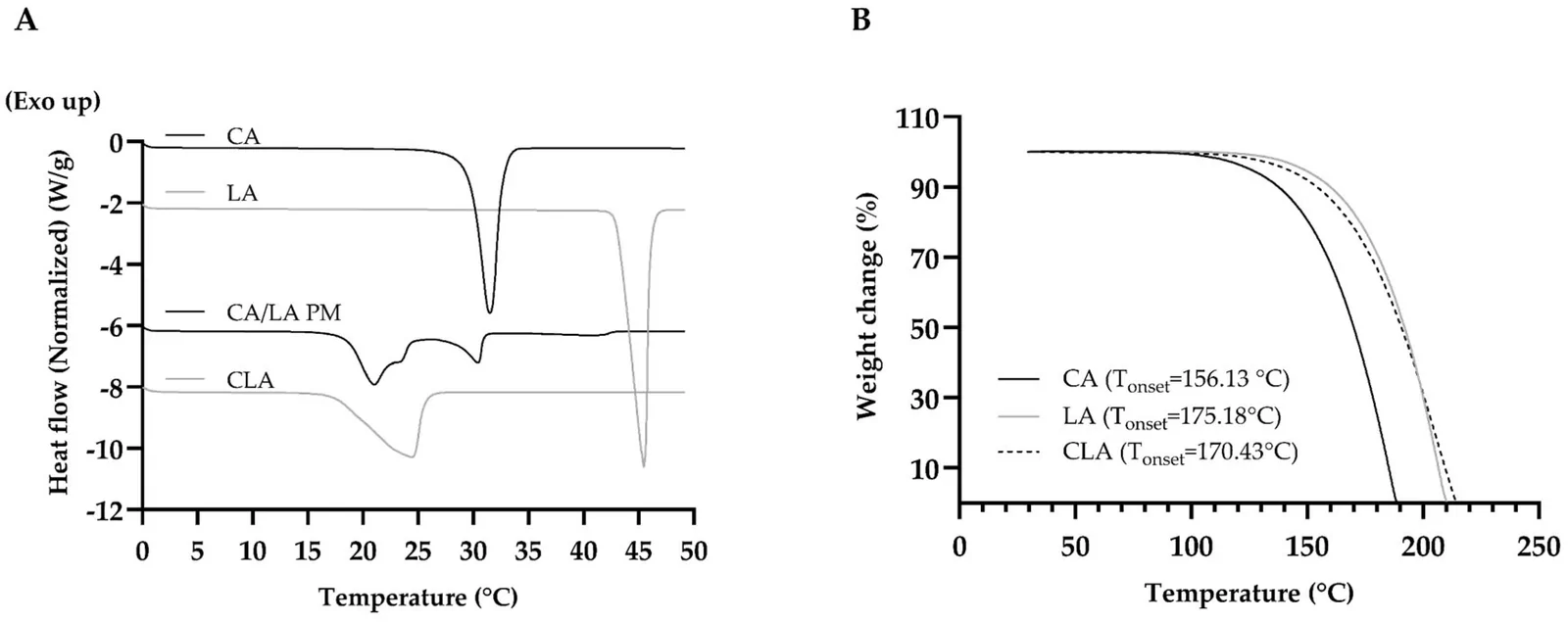
(**A**) DSC thermograms of CA, LA, CA/LA PM, and CLA. (**B**) TGA thermograms of CA, LA, and CLA. CA: capric acid; LA: lauric acid; CA/LA PM: physical mixture of capric acid and lauric acid; CLA: capric acid–lauric acid 2:1.

In addition to FT-IR analysis, DSC was performed to further confirm the interactions between CA and LA and the formation of the eutectic solvent. As shown in Figure 4A, the thermograms of the pure components exhibited single, well-defined endothermic peaks, with onset temperatures of 29.71 °C and 31.50 °C for CA and LA, respectively. The thermogram of the physical mixture of CA and LA (CA/LA PM) exhibited two endothermic peaks with lower melting temperatures (onset temperatures of 18.60 °C and 28.17 °C) and reduced peak intensities compared to the pure fatty acids. The decrease in peak intensity is consistent with the molar proportions of CA and LA in the mixture, indicating that each component influenced the crystallization and melting behavior of the other. Although the DSC data do not allow definitive identification of the component that initiated melting, the observed melting point depression indicates eutectic interactions between the fatty acids induced by thermal treatment during DSC analysis. The thermogram of the CLA mixture displayed a single endothermic peak with an onset temperature of 17.35 °C, which was substantially lower than those of the individual fatty acids. These results demonstrate reduced melting heat flow and altered thermal behavior in CLA compared to the pure components (Figure 4). This pronounced melting point depression is characteristic of eutectic systems and has previously been reported for binary mixtures of consecutive saturated fatty acids [19,43]. According to the literature, the investigated CA–LA molar ratio (2:1) is close to the eutectic composition, which explains the significantly lower melting transition observed for the mixture compared to the pure components [44,45]. The presence of a single melting event in CLA provides strong evidence that a eutectic phase was formed rather than a simple physical blend of two fatty acids.

Assessing the thermal stability of a eutectic solvent is essential for defining the temperature range within which it can be used without undergoing structural or compositional changes. Figure 4B shows the thermogravimetric profile of CLA and its individual constituents, illustrating the variation in sample mass as a function of temperature and enabling the determination of their respective degradation temperatures.

The thermogravimetric profiles demonstrated that CA, LA, and CLA underwent pronounced thermal degradation within a relatively narrow temperature range, although their degradation behaviors differ substantially. The onset of mass loss for CA occurred at approximately 156.13 °C and extended to 184.38 °C, corresponding to a mass loss of about 98.94%. LA began to degrade at a higher temperature, approximately 175.18 °C, with degradation continuing to 202.47 °C and producing a mass loss of 100%. By contrast, CLA prepared from CA and LA at a 2:1 molar ratio exhibited an onset temperature of approximately 170.43 °C and reached the end of the principal degradation event at 206.07 °C, with a complete mass loss. The onset temperature of CLA lies between those of the two pure components and is considerably higher than that of CA, indicating that the eutectic system is thermally more stable than CA alone, although slightly less stable at the onset of degradation than LA. In practical terms, TGA shows that CLA appears to remain thermally usable up to approximately 170 °C, although prolonged operation should preferably be conducted well below this temperature to minimize degradation that could reduce extraction reproducibility and potentially compromise the chemical stability of thermolabile target compounds.

Collectively, the FT-IR, DSC, and TGA results demonstrate that the CLA system shows spectral and thermal properties that differ from those of the individual components, which is consistent with the formation of a eutectic system. Nevertheless, the described techniques were used primarily as complementary characterization methods, and the obtained data cannot by themselves establish the detailed molecular mechanism of eutectic formation.

The significantly higher efficiency of CLA in comparison to other investigated DESs may be explained by its highly hydrophobic and lipid-like character, which is particularly compatible with the predominantly lipophilic nature of carotenoids. The combination of medium- and long-chain fatty acids can disrupt the crystal packing of individual components, decrease the melting point, and generate a more disordered eutectic phase with improved solvation capacity [46].

**Figure 5:**
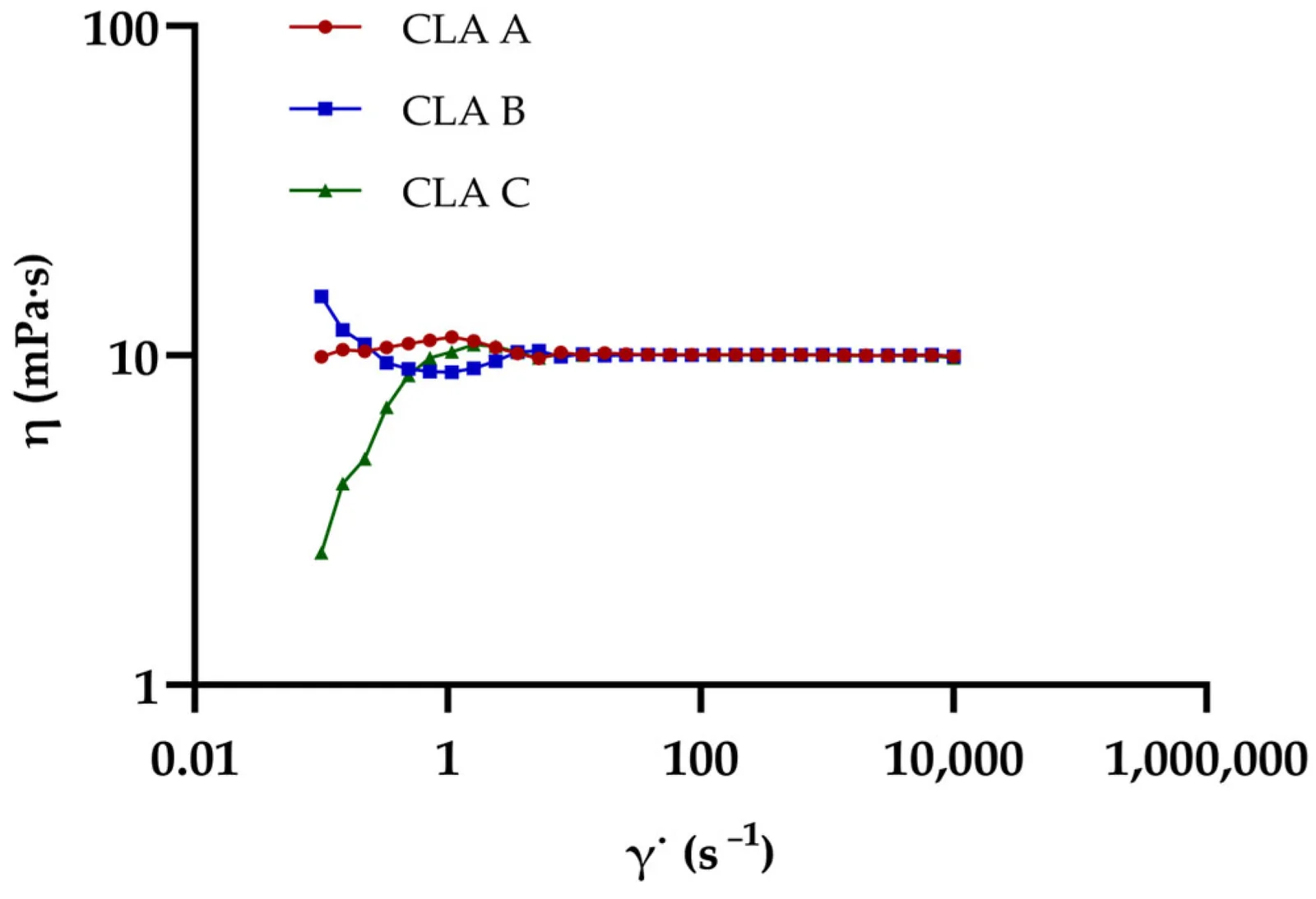
Flow curve of CLA measured at 25 °C over the investigated shear-rate range. CLA: capric acid: lauric acid 2:1. A, B, and C represent three consecutive measurements. CLA: capric acid–lauric acid 2:1.

The rheological properties of DESs are influenced by several factors, including the nature and molar ratios of the constituent components, temperature, and water content. Although DESs are recognized as environmentally friendly solvents and promising alternatives to conventional organic solvents, most of them are characterized by high viscosities, which can restrict mass transfer and consequently reduce the solvent’s extraction efficiency [47]. As shown in Figure 5, CLA exhibited a viscosity of 9–11 mPa·s at 25 °C, which remained nearly constant across the examined shear-rate range, indicating predominantly Newtonian behavior. These values place CLA among low-viscosity DES, considering that the viscosity of most DESs exceeds 100 mPa·s at 20 °C. Such rheological properties are advantageous for extraction processes, as low viscosity facilitates particle wetting, solvent penetration, and mass transfer between the tomato pomace matrix and the extraction medium [47]. Due to its hydrophobic nature and its composition based on fatty acids, CLA provides a favorable environment for the solubilization of lipophilic carotenoids. Furthermore, under MAE conditions, solvent heating is expected to further reduce viscosity and enhance diffusion rates, thereby promoting carotenoid release from disrupted cellular structures and improving extraction efficiency [16,48,49,50]. By contrast, MLA contains lactic acid, which introduces greater polarity and hydrogen-bonding capacity. High hydrogen-bonding capacity can increase viscosity and limit diffusion, while greater polarity increases interactions with polar matrix constituents and limits carotenoid-selective partitioning. Although MT is also hydrophobic, the menthol–thymol system exhibited less favorable solvent–matrix and solvent–carotenoid interactions under the applied MAE conditions [46].

Taking into account the yields of BC and LYC obtained under both MAE conditions (Figure 2), SO and CLA were used in further steps of MAE optimization.

#### 3.1.2. Determination of Solid-to-Solvent Ratio

Available literature data (Table 1) show a wide range of solid-to-solvent ratios applied in green protocols for the extraction of carotenoids from TPW, ranging from 1:10 (*w*/*v*) up to 1:140 (*w*/*v*). The solid-to-solvent ratio is a critical parameter in developing green extraction procedures for carotenoids because it directly governs the extraction efficiency, solvent consumption, and overall environmental footprint of the process. An optimal ratio maximizes the driving force for mass transfer and carotenoid yield while minimizing the volume of solvent required, which is essential for reducing waste, energy demand for solvent recovery, and process costs in line with green chemistry principles. Conversely, too low a ratio can lead to saturation and poor recovery, whereas too high a ratio unnecessarily increases solvent use and downstream processing burdens, undermining the sustainability goals of the method.

As presented in Table 4, changes in the mass-to-solvent ratio had no significant effect on BC extraction yields but influenced yields of LYC. In the case of SO, the optimal mass-to-solvent ratio was 1:10 because it provided significantly higher yields of LYC (and consequently total carotenoids) compared to 1:20 and 1:30 ratios. This can be explained by the fact that in MAE, the sample itself acts as a susceptor: with less solvent (1:10), the same microwave power is deposited into a smaller thermal mass, producing faster and higher local temperatures, stronger cell wall rupture, and more efficient release of LYC per unit of dry matter. At a 1:20 ratio, the larger solvent volume absorbs and dissipates more microwave energy, lowering the effective heating rate of the matrix and reducing the extent of cell disruption at the same time, so less LYC is liberated despite the larger solvent reservoir. This is in line with the results of Ho and coworkers, who investigated the MAE of LYC using ethyl acetate and obtained maximal yields with a 1:10 mass-to-solvent ratio [13]. Also, other authors showed that low mass-to-solvent ratios (8–10:1) are efficient for the MAE of carotenoids using vegetable oils as extraction solvents [48]. By contrast, CLA is much more polar and often contains ionizable carboxylic groups that give it a higher dielectric loss factor and better microwave coupling compared to vegetable oils. Therefore, it heats efficiently under microwave radiation, and its viscosity and solvation power for carotenoids improve markedly with temperature. At a 1:20 ratio, the larger DES volume can probably absorb more microwave energy, maintaining a higher bulk temperature and providing a stronger solvation driving force for LYC, whereas the smaller DES volume may reach a high local temperature fast but then be limited by a lower total solvent capacity [49]. Based on the obtained data, further optimization of MAE was conducted at a 1:10 mass-to-solvent mass ratio for SO and a 1:20 mass-to-solvent mass ratio for CLA.

### 3.2. Optimization of Carotenoid Extraction

MAE optimization was performed using two different solvents independently—SO and CLA. The experimental points were selected to cover mild, intermediate, and intensive microwave treatments and to identify both performance-enhancing and potentially degradative conditions. For SO, the investigated range was sufficient to reveal a decline in carotenoid recovery under excessive treatment. For CLA, the initial range did not identify a clear upper performance boundary; therefore, additional higher power and longer time conditions were introduced sequentially. This adaptive procedure was used to define the solvent-specific temperature/power range.

The results of SO extraction are presented in Table 5. The yields of LYC (mg/100 g) ranged from a minimum of 1.53 ± 0.30 (13 min, 1600 W) to a maximum of 4.10 ± 0.04 (7 min, 700 W), and the yields of BC (mg/100 g) ranged from 2.44 ± 0.22 (13 min, 1600 W) to 3.17 ± 0.18 (1 min, 1600 W). Evidently, MAE had a stronger impact on the release of LYC from cellular compartments. This can be attributed to the enhanced extractability of lycopene using MAE, but also to the generally higher abundance of LYC (compared to BC) in TPW. The extreme conditions led to a decreased yield of both carotenoids, indicating that the limits of the extraction window were identified. The selection of optimized conditions was achieved by identifying the extraction runs characterized by an optimal ratio of obtained yield and energy expenditure. Finally, the selected extraction parameters were 4 min and 217 W, resulting in 3.57 ± 0.27 mg/100 g of LYC, 2.82 ± 0.24 mg/100 g of BC, and 0.014 kWh of energy consumption.

A contrasting response was seen in the case of DES extraction (Table 6). Here, the initial border conditions did not lead to a reduction in the yield; therefore, the extraction space was expanded by including more extreme borderline conditions (as described in the materials and methods section). This strongly indicates that DESs can sustain harsher extraction conditions compared to SO without resulting in carotenoid degradation. LYC yields ranged from 2.54 ± 0.11 mg/100 g (1 min, 100 W) to 9.80 ± 1.77 mg/100 g (13 min, 1800 W). Similarly, BC yields spanned from 2.61 ± 0.11 mg/100 g to 5.29 ± 0.84 mg/100 g under the same MAE conditions. The highest yields of both LYC and BC were obtained at 13 min and 1800 W, which were identified as the optimal extraction conditions. Under these conditions, LYC and BC yields reached 9.80 ± 1.77 mg/100 g and 5.29 ± 0.84 mg/100 g, respectively, with an energy consumption of 0.390 kWh.

The fact that CLA coupled with stronger microwave power led to increased yield (compared to SO) indicates its compatibility for utilization in MAE. The observed performance of DESs may be attributed to their low volatility, thermal stability, and favorable interactions with hydrophobic carotenoids. These properties facilitate carotenoid solubilization while maintaining solvent performance under intensive microwave treatment.

The findings of the current study on the thermal stability and extraction efficiency of DESs are consistent with earlier reports that examined their use as environmentally friendly solvents for carotenoid recovery. For example, Silva and co-authors [19] investigated the extraction efficiency of a eutectic solvent composed of DL-menthol and lactic acid at an 8:1 molar ratio for LYC recovery from tomato processing byproducts using ultrasound-assisted extraction (UAE). They reported yields of 1446.6 μg/g d.w., which were higher than those obtained using ethyl acetate and ethyl lactate (1334.8 μg/g d.w.). Similarly, Marinaccio and co-workers [18] examined the efficacy of a eutectic mixture of DL-menthol and thymol at a 1:1 molar ratio for LYC extraction from tomato skin waste, also using ultrasound-assisted extraction. The results demonstrated that the menthol–thymol mixture exhibited good extraction efficiency compared to hexane as a conventional solvent, yielding 484.2 ± 2.4 mg/g DE and 735.9 ± 8.8 mg/g DE, respectively. Similar findings were reported by Vlachoudi and co-authors [15], who investigated the extraction efficiency of menthol in combination with hexanoic and octanoic acids, as well as their combined use at several molar ratios, and compared these mixtures with ethyl acetate, hexane, and acetone. The study showed that the menthol–hexanoic acid mixture at a 2:1 molar ratio exhibited significantly higher extraction efficiency than the other mixtures and solvents evaluated, including ethyl acetate, hexane, and acetone. These studies strongly indicate that eutectic solvents are good alternatives to conventional solvents for carotenoid recovery from agro-industrial byproducts such as TPW.

Edible oils, including SO, have proven to be effective green solvents for carotenoid extraction and good alternatives to commonly used conventional solvents [11], especially due to their lipophilic nature. Ultrasound-assisted extraction of carotenoids from fresh carrots using SO as the extraction solvent resulted in similar yields of BC (334.75 mg/L) after 20 min of extraction compared to hexane (321.35 mg/L) after 20 min of conventional solvent extraction (CSE) [50]. Based on the available literature data and comparison of the obtained yields, it can be concluded that several factors affect carotenoid yield: the extraction temperature, the power of the ultrasonic or microwave treatment, and the stirring speed in conventional extraction. For example, Nour and co-workers evaluated SO efficiency using MAE (5 min, 700 W). The obtained result was 32.2 ± 1.3 mg/kg of total carotenoids, which is a significantly lower yield than the results obtained in this research under optimal MAE conditions (4 min, 217 W) [51].

Despite the good yields and extraction efficiency reported, SO showed lower potency in carotenoid extraction compared to DESs. This could be explained by the higher viscosity of SO, which can limit solvent penetration into the plant matrix and decrease mass transfer during extraction. Hydrophobic DESs, on the other hand, exhibit strong affinity for nonpolar carotenoids and lower viscosity than most conventional DES formulations, facilitating mass transfer and improving extraction efficiency during MAE [23,52]. These properties make them suitable solvents for carotenoid extraction, especially for nonpolar compounds from food matrices [53]. Additionally, compared to many standard extraction media, DESs typically exhibit greater microwave absorption. This could lead to more effective heating and improved plant structure disruption during MAE, which would facilitate carotenoid release [54]. This hypothesis is consistent with the current study’s results, which showed that CLA consistently produced higher yields of LYC and BC than SO, especially when MAE conditions were intensified.

To the best of our knowledge, no studies have reported the optimization of MAE for carotenoid extraction using SO and CLA as extraction solvents. Therefore, the present findings are discussed in relation to studies employing comparable green solvents or alternative extraction techniques. Overall, currently available literature data show that the extraction method and solvent selection are important factors for the recovery of carotenoids derived from tomato byproducts. Studies using hydrophobic DESs consistently report extraction efficiencies comparable to or even higher than those obtained using conventional organic solvents, highlighting their applicability for the recovery of lipophilic compounds such as LYC and BC [16,24,55,56]. Furthermore, recent studies show that extraction methods such as MAE and UAE improve carotenoid extraction by reducing the extraction time. Similar trends reported for other green solvents, such as ethanol, ethyl acetate, and ethyl lactate, confirm the increasing potential of sustainable extraction systems for the valorization of tomato processing byproducts [14,20,57,58].

The DES based on capric acid and lauric acid investigated in this research paper achieved substantially higher carotenoid recoveries than SO, particularly under MAE conditions with high microwave power and extended extraction times. Although direct comparison with literature data is limited by the lack of studies on the investigated extraction solvents using MAE, as well as differences in raw material characteristics, drying techniques, homogenization and milling conditions, particle size, solid-to-solvent ratio, and whether results are expressed on a fresh or dry sample basis, this research highlights the potential of hydrophobic DESs, especially CLA, as green solvents for future investigations of carotenoid extraction from TPW.

### 3.3. Impact of TPW Pretreatment by Vibrational Micro-Milling

Particle size reduction is an important step in optimizing carotenoid extraction because it increases the specific surface area available for solvent contact, shortens the diffusion path, and facilitates the disruption of plant tissues and chromoplast structures in which carotenoids are localized. These effects can improve solvent penetration and mass transfer, thereby increasing the recoveries of LYC and BC. On the other hand, excessively fine milling may promote agglomeration, hinder solvent flow, or accelerate carotenoid degradation through heat, light, and oxygen exposure; therefore, particle size and the milling conditions should be optimized together with the extraction solvent and process parameters [1]. Vibrational micro-milling offers specific advantages over conventional grinding because high-frequency oscillatory impacts can produce more uniform and efficient comminution, including the rupture of rigid cell structures and improved homogenization of raw material. By increasing the accessibility of carotenoids to the extraction medium, pretreatment in a vibrational micro-mill may therefore increase carotenoid yields, reduce extraction time or solvent requirements, and improve process reproducibility. However, milling should be performed under controlled frequency, duration, and temperature, with limited exposure to oxygen and light, to maximize cell disruption while minimizing carotenoid oxidation and isomerization.

Vibrational micro-milling significantly decreased the particle size of TPW, and all presented parameters were 62–78% lower in pretreated TPW. The median particle size, D50, decreased from approximately 303 µm to 66.8 µm, indicating that the typical particle after vibrational micro-milling was about 4.5 times smaller. The particularly significant D90 reduction (from 849 to 191 µm) further shows that VM was especially effective in reducing the coarse particle fraction. The volume-weighted mean diameter decreased from 382.2 ± 20.8 to 122.8 ± 12.8 µm, and the surface-area-weighted mean diameter, D, decreased from 102.3 ± 3.41 to 38.1 ± 0.2 µm. The difference between the two values was smaller in VM, suggesting that VM generated a somewhat more homogeneous distribution, although the distribution remained polydisperse.

**Figure 6:**
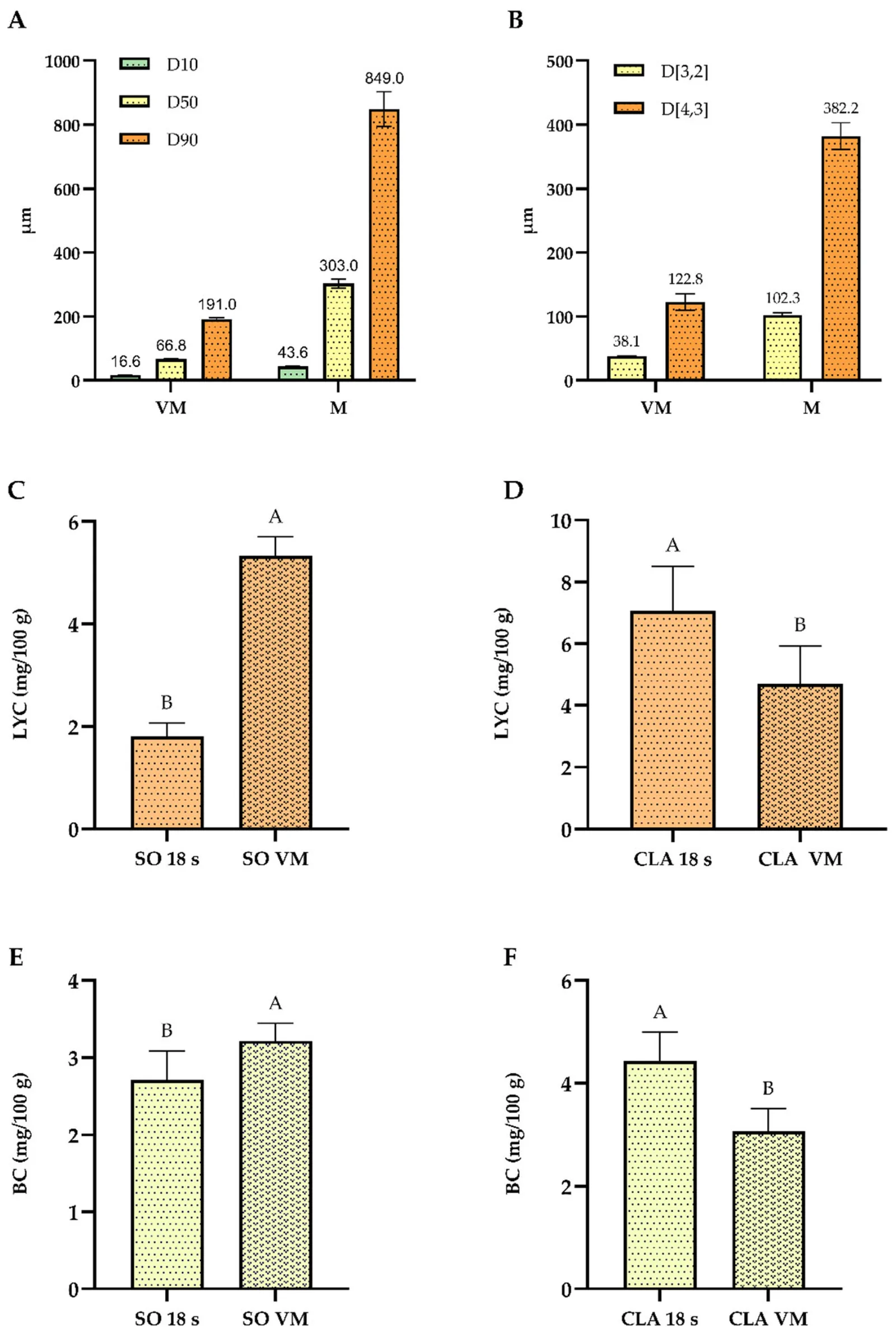
Comparison of milling (M) and vibrational micro-milling (VM) in terms of particle size and distribution (**A**,**B**); LYC yields (**C**,**D**) and BC yields (**E**,**F**). Results are shown as mean ± SD. Statistical differences between the two samples were evaluated using Student’s *t*-test. Different letters above the bars indicate significant differences (*p* < 0.05) within each measured component. SO 18 s: sunflower oil extract of TPW milled for 18 s; SO VM: sunflower oil extract of TPW additionally treated using a vibrating micro-mill; CLA 18 s: extract of TPW in a capric acid–lauric acid 2:1, milled for 18 s; CLA VM: extract of TPW in a capric acid–lauric acid 2:1, additionally treated using a vibrating micro-mill.

Particle size reduction had the opposite effects in SO compared to CLA in terms of BC and LYC yields (Figure 6C–F). The obtained results might be explained by the fact that micro-milling increases the specific surface area, reduces intraparticle diffusion distances, and disrupts cellular structures, thereby improving solvent penetration and the release of matrix-bound carotenoids. Under the relatively mild MAE conditions used for SO (217 W, 4 min), these mass-transfer advantages predominated, resulting in increased BC and LYC yields. Conversely, under the substantially harsher MAE conditions applied for CLA (1800 W, 13 min), the finely milled material likely exhibited greater susceptibility to rapid microwave heating and carotenoid degradation. The increased exposed surface area and disruption of the protective matrix may have facilitated the thermal and oxidative degradation, and possibly isomerization, of the released carotenoids. Therefore, under severe MAE conditions, degradative losses apparently exceeded the benefits of enhanced carotenoid release, leading to lower carotenoid contents in the micro-milled samples.

Previous studies have shown that particle size reduction can enhance carotenoid recovery by increasing the specific surface area, reducing diffusion distances, and disrupting cell wall barriers. For example, jet milling of Chlorella powder from 67 to 20 µm increased lutein saponification and extraction 2.3–2.9-fold, while reduction of tomato pomace particle size from <2 mm to <0.5 mm increased LYC recovery approximately 1.6-fold under supercritical fluid extraction at 50 °C. However, the relationship is not necessarily linear: optimization of carotenoid extraction from tomato waste identified an intermediate particle size of 0.56 mm as optimal, demonstrating that the effect of comminution depends on the extraction medium and operating conditions [59]. Tagliasco and co-authors investigated the effect of milling and thermal sample pretreatment on LYC yields in supercritical fluid extraction, reporting that the effect of particle size reduction on LYC yields depended significantly on the sample pretreatment conditions: it was positive at lower temperatures and negative for higher thermal pretreatment temperatures [60]. Accordingly, the divergent effects observed in SO and CLA may reflect a shift from mass-transfer-limited extraction under mild MAE conditions to degradation-limited extraction under the more severe conditions applied with CLA.

### 3.4. Comparison of Green Extraction Approaches—MAE vs. CSE and UAE

**Figure 7:**
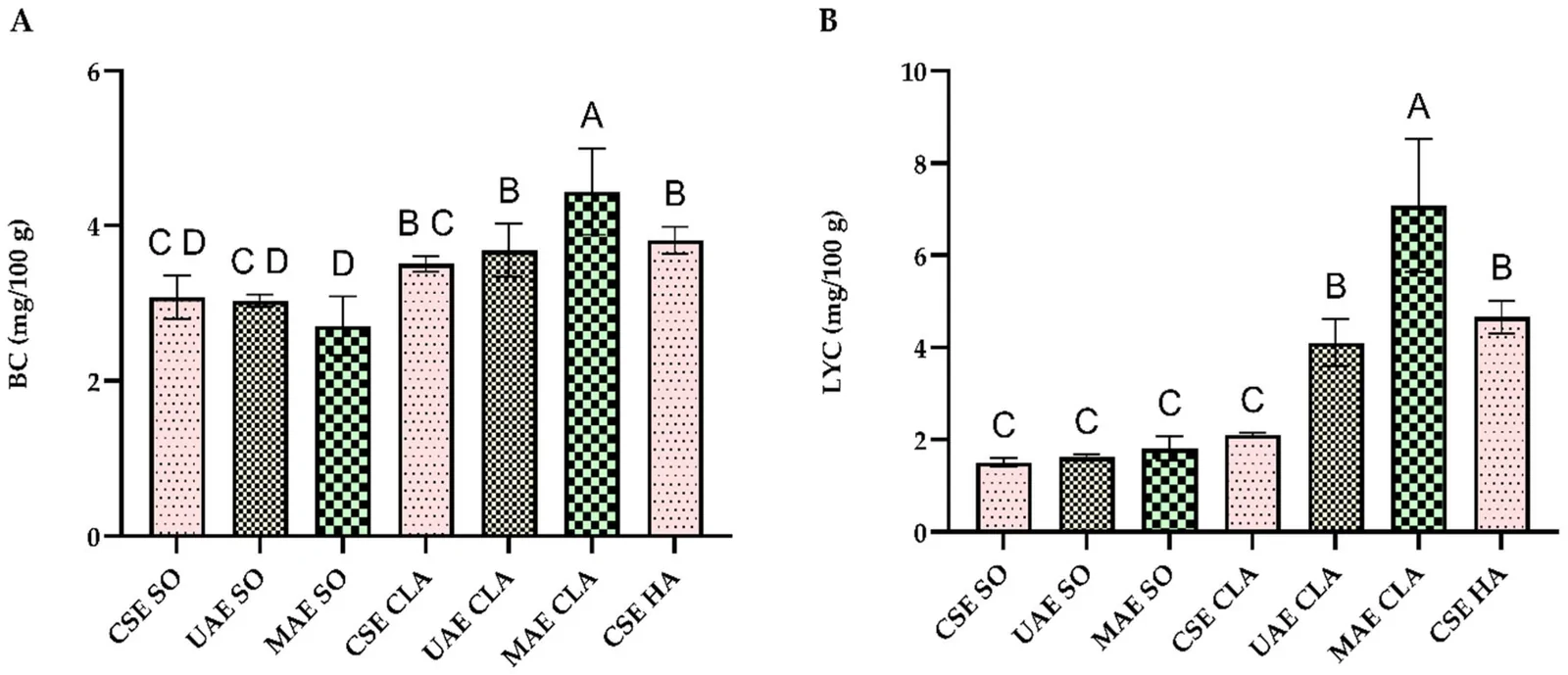
(**A**) BC and (**B**) LYC yields depending on the extraction technique and the applied extraction solvent. Results are shown as mean ± SD. Statistical significance was evaluated using one-way ANOVA with Tukey’s post hoc test. Different letters above the bars indicate significant differences (*p* < 0.05) within each measured component. CSE SO: conventional solvent extraction using sunflower oil; UAE SO: ultrasound-assisted extraction using sunflower oil; MAE SO: microwave-assisted extraction using sunflower oil; CSE CLA: conventional solvent extraction using capric acid–lauric acid 2:1; UAE CLA: ultrasound-assisted extraction using capric acid–lauric acid 2:1; MAE CLA: microwave-assisted extraction using capric acid–lauric acid 2:1; CSE HA: conventional solvent extraction using hexane–acetone 1:1.

To critically evaluate the developed MAE for BC and LYC, the extraction yields obtained under optimized conditions with SO and CLA were compared to yields obtained under optimized CSE and UAE conditions reported by other authors [16,18] using SO and CLA (CSE, UAE) and an HA 1:1 (CSE) mixture as the extraction solvents. The HA mixture is often selected as the conventional reference extraction system because it is a well-established, broadly effective, and experimentally reproducible solvent system for recovering carotenoids and other lipophilic compounds [6]. The obtained data are presented in Figure 7A,B.

The BC values ranged from 2.71 ± 0.38 in MAE-SO up to 4.44 ± 0.56 in MAE-CLA. CLA was also applicable in CSE and UAE, providing yields significantly lower than MAE but comparable to HA extracts obtained using CSE. Even though it is used frequently as a food-grade solvent for carotenoids, SO provided the lowest BC yields among the investigated solvents, regardless of the applied extraction technique. Similar trends were observed for LYC, with more pronounced differences among the applied extraction techniques.

Again, MAE using CLA as the extraction solvent provided the highest LYC yields (7.07 ± 1.44 mg/100 g), followed by UAE using CLA as the extraction solvent and CSE using the HA mixture, providing significantly lower, comparable LYC yields (4.10 ± 0.52 and 4.66 ± 0.36 mg/100 g respectively). The lowest yields were obtained in SO, where MAE was shown to be somewhat superior to CSE and UAE. Overall, MAE using CLA consistently provided the highest BC and LYC yields, whereas SO resulted in the lowest yields irrespective of the extraction technique applied. However, as stated previously, extraction efficiency should not be evaluated solely based on compound yield, as solvent toxicity, energy consumption, resource use, and associated environmental impacts are also important considerations; therefore, the investigated extraction procedures were further compared using an LCA. The results of the selected environmental footprints of the seven extraction methods are presented in Table 7.

**Figure 8:**
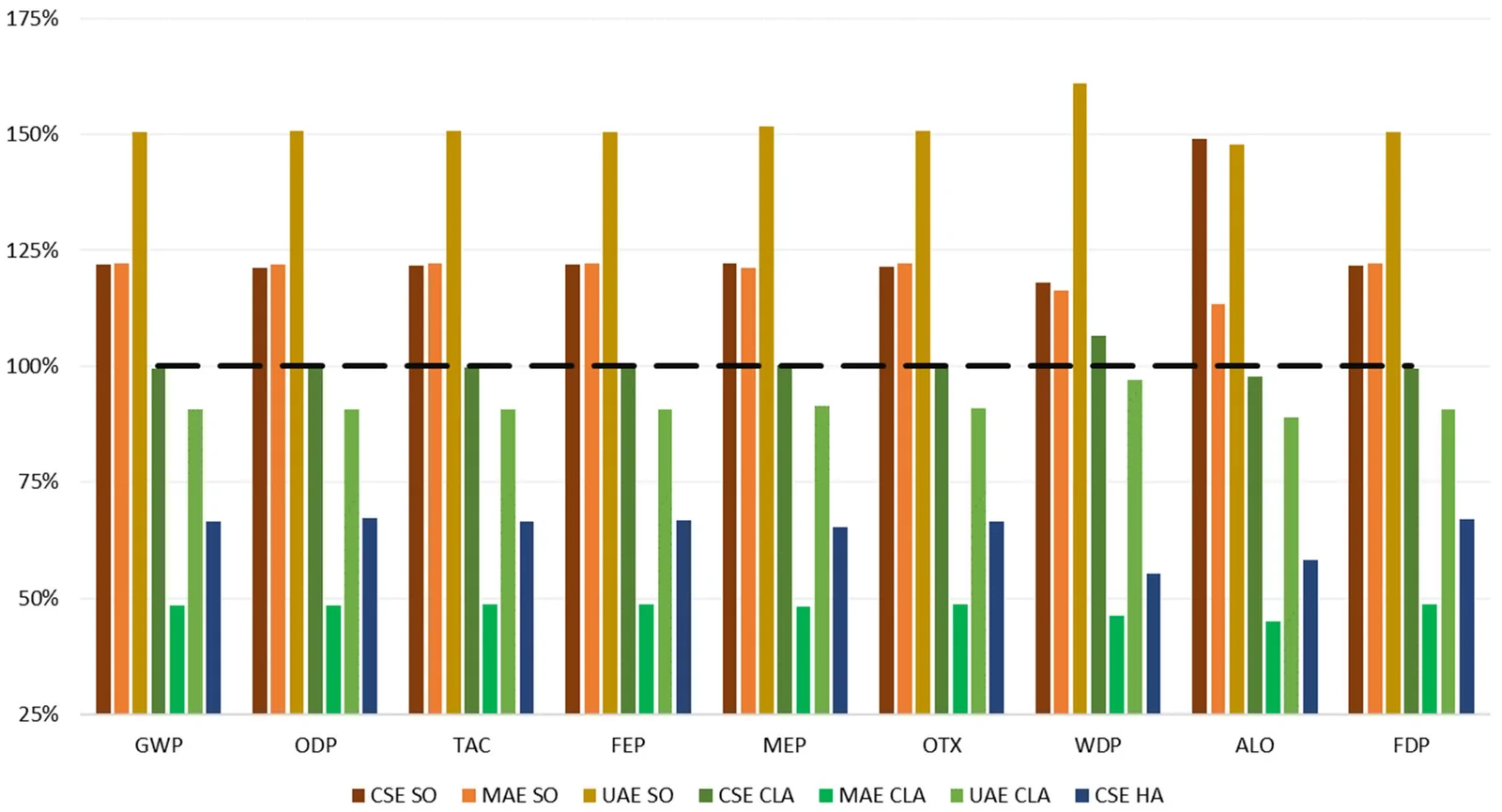
Comparison of relative values of environmental footprints for all seven treatments. The dashed line indicates the reference level (100%), corresponding to the mean value across all extraction methods within each environmental impact category. CSE SO: conventional solvent extraction using sunflower oil; UAE SO: ultrasound-assisted extraction using sunflower oil; MAE SO: microwave-assisted extraction using sunflower oil; CSE CLA: conventional solvent extraction using capric acid–lauric acid 2:1; UAE CLA: ultrasound-assisted extraction using capric acid–lauric acid 2:1; MAE CLA: microwave-assisted extraction using capric acid–lauric acid 2:1; CSE HA: conventional solvent extraction using hexane–acetone 1:1; GWP: global warming potential; ODP: ozone depletion potential; TAC: terrestrial acidification; FEP: freshwater eutrophication; MEP: marine eutrophication; OTX: overall toxicity; HTX: human toxicity; FTX: freshwater ecotoxicity; MTX: marine ecotoxicity; TTX: terrestrial ecotoxicity; WDP: water depletion; FDP: fossil fuel depletion; ALO: agricultural land occupation.

Based on the inventory, Table 7 shows that when SO was used, microwave-assisted extraction (MAE) exhibited the lowest environmental impacts across all selected categories. When CLA was used, conventional extraction (CE) showed the most favorable environmental performance for most impact categories. However, because carotenoid recovery was the primary objective of the study, the environmental impacts were also evaluated in relation to extraction performance. Accordingly, Figure 8 presents the relative environmental impacts, expressed as percentages of the corresponding mean values used as the reference point (100%). When interpreted together with the carotenoid yields, the results indicate that MAE using CLA provided the most favorable overall balance between extraction efficiency and environmental performance, followed by conventional extraction using the HA mixture. These findings demonstrate that the environmental sustainability of an extraction protocol cannot be inferred solely from the use of a food-grade solvent or an unconventional extraction technology; rather, it must be quantitatively assessed using a life-cycle perspective and interpreted alongside the amount and quality of the target compounds recovered. An LCA is therefore essential for identifying environmental trade-offs and determining whether an apparently “green” extraction method provides a genuine improvement over conventional alternatives.

## 4. Conclusions

This study demonstrated the potential of MAE for recovering BC and LYC from TPW. Among the solvents evaluated, SO and CLA provided the highest carotenoid yields. The optimal MAE conditions depended on the solvent. For SO, the selected conditions were 217 W for 4 min, yielding 2.82 ± 0.24 mg/100 g of β-carotene and 3.57 ± 0.27 mg/100 g of lycopene (0.014 kWh). For CLA, the selected conditions were 1800 W for 13 min, resulting in 5.29 ± 0.84 mg/100 g of β-carotene and 9.80 ± 1.77 mg/100 g of lycopene (0.390 kWh). Thus, CLA provided higher carotenoid recoveries than SO, particularly under intensive MAE conditions, although with higher energy consumption. The effect of vibrational micro-milling on carotenoid recovery depended on the extraction medium and MAE conditions: micro-milling improved yields in SO under mild conditions but reduced yields in CLA under more intensive conditions, indicating that increased matrix disruption can promote degradation when carotenoids are exposed to excessive thermal stress.

Compared to CSE and UAE, MAE-CLA provided the highest recovery of both target carotenoids. The partial gate-to-gate LCA showed that MAE-SO had the lowest impacts among SO-based treatments, whereas CSE-CLA was most favorable in most individual impact categories. When environmental impacts were considered relative to carotenoid recovery, MAE using CLA provided the most favorable overall balance between extraction efficiency and environmental performance.

The most important limitations of the study are the fact that the experiments were performed at the laboratory scale using TPW from a limited number of production years and one source; therefore, seasonal, varietal, and industrial variability were not fully assessed. CSE and UAE conditions were adopted from previous studies rather than optimized under the same experimental design as MAE, and an organoleptic evaluation of the obtained extracts was not conducted. Furthermore, the LCA was a partial gate-to-gate assessment that did not include TPW production, upstream transport, solvent recovery, product purification, or scale-up, and the environmental profile may change under different electricity mixes or industrial operating conditions.

Future research should validate the optimized MAE conditions using larger and more representative batches of TPW and assess their robustness across tomato cultivars, processing conditions, and storage periods. The recyclability of CLA, the recovery and stability of the extracted carotenoids, and the feasibility of downstream formulations should also be investigated. Finally, pilot-scale experiments combined with an expanded LCA, including solvent recovery, process integration, scale-up, and different regional energy scenarios, will be necessary to determine whether the proposed CLA-based MAE process can provide a demonstrable environmental and techno-economic advantage for industrial TPW valorization.

## Figures and Tables

**Table 1:** Protocols for extracting carotenoids from tomato waste using DESs.

Solvent	Solvent Preparation	Extraction Technique	Protocol	Detection	Reference
Menthol–hexanoic acid 2:1; 1:1	Menthol and fatty acids were mixed and heated at 70 °C under stirring at 350 rpm until a clear homogeneous liquid was formed. The solvents were cooled to room temperature and inspected for the formation of crystals after 24 h.	CSE	1:10 (*w*/*v*), 500 rpm, 60 min, room temperature	Total carotenoids (UV–Vis)	[15]
Menthol–octanoic acid 1:1					
Hexanoic acid–octanoic acid 2:1					
Capric acid–lauric acid 1:2	The mixtures were heated at 50 °C upon stirring (750 rpm) until transparent homogeneous liquids were obtained.	CSE	1:40 (*w*/*v*), 750 rpm, 60 min, room temperature	Lycopene (HPLC); TC (UV–Vis)	[16]
Thymol-lauric acid 1:2					
Thymol–lauric acid 2:1					
DL-menthol–lactic acid 8:1	Lactic acid was added to menthol. The mixtures were heated using a magnetic stirring heating plate at 1200 rpm until no solid particles remained.	UAE CSE	UAE (1:120 (*w*/*v*), 70 °C,10 min, 40 kHz, 100 W); CSE (3.4:20 (*w*/*v*), 50 °C, 1.5 h water bath)	Lycopene (UV–Vis)	[17]
Thymol–menthol 1:1	An equimolar solution of thymol: menthol (1:1) was prepared by adding 10.0 g of menthol to 9.6 g of thymol in a round bottom flask, shaking with a magnet at room temperature for 1 h to obtain a total volume of 20 mL.	UAE	1:20 (*w*/*v*), 36 °C, 20 min	Lycopene (HPLC)	[18]
DL-menthol–lactic acid 8:1	Lactic acid was added to menthol. The mixtures were heated using a magnetic stirring heating plate at 1200 rpm until no solid particles remained.	UAE	1:120 (*w*/*v*), 70 °C, 10 min, 40 kHz, 100 W	Lycopene (UV–Vis)	[19,20]
DL-menthol–butanoic acid 1:1, 1:2, 2:1	The components were placed in a beaker, heated to 50 °C, and stirred at 200 rpm for 30 min until the solution became liquid and transparent.	CSE	1:10 (*w*/*v*), room temperature 24 h, shaking incubator (450 rpm)	Lycopene, β-carotene (HPLC)	[21]
DL-menthol–hexanoic acid 1:1, 1:2, 2:1					
DL-menthol–caprylic acid 1:1, 1:2, 2:1					
DL-menthol–lauric acid 1:1, 1:2, 2:1					
DL-menthol–camphor 1:1	The components were added together, and the mixture was stirred continuously for 2 h at 50 °C until a clear and homogeneous liquid formed.	PLE UAE	1:12.5 (*w*/*v*), 640 W, 35 kHz, 30 min, 25 °C	Lycopene, β-carotene (HPLC)	[22]
Octanoic acid–decanoic acid 1.8:1	The components were mixed in the defined molar ratios, followed by heating at 80 °C for 30 min under constant stirring (1000 rpm) until a homogeneous liquid phase was obtained.	CSE	1:10 (*w*/*w*), 45 °C, 900 rpm, 120 min	(All-E)-lycopene, β-carotene, Lutein, Z-lycopene (HPLC); Total carotenoids (UV–Vis)	[23]
Choline chloride–1,3-butanediol 1:5	The mixtures were heated to between 50 °C and 100 °C using a magnetic stirring heating plate at 900 rpm for 1–4 h until a transparent, homogeneous liquid was obtained.	UAE	1:20 (*w*/*v*), 12 min, 65 °C, 37 Hz, ultrasound power level of 70%	Lutein, β-carotene, Lycopene	[24]

CSE: conventional solvent extraction; UAE: ultrasound-assisted extraction; MAE: microwave assisted extraction; HPLC: high-performance liquid chromatography; UV–Vis: ultraviolet–visible spectroscopy.

**Table 2:** Composition and preparation of DESs.

Components	Ratio	Preparation	Characteristics
**Menthol–thymol**	1:1	Dissolve each component at 51 °C (water bath) and 120 rpm (until fully melted). Transfer each component to a preheated magnetic stirrer at 51 °C and 200 rpm and mix for 10 min. Combine and mix for an additional 10–15 min until a homogeneous, transparent liquid forms.	Stable at RT.
**Menthol–lactic acid**	8:1		Unstable. DES not formed.
	4:1		Stable at RT.
	2:1		Unstable. DES not formed.
	1:1		Unstable. DES not formed.
**Capric acid–lauric acid**	1:2		Unstable. DES not formed.
	2:1		Stable at RT.

DES: deep eutectic solvent; RT: room temperature. Shaded cells indicate solvents that were stable at room temperature and were selected for further investigation.

**Table 3:** Input variable levels adopted in the MAE optimization.

	SO		CLA	
Input Variables	Min	Max	Min	Max
Microwave power (W)	100	1300	100	1800
Extraction time (min)	1	13	1	25

SO: sunflower oil; CLA: capric acid–lauric acid 2:1.

**Table 4:** Yields of BC, LYC, and total carotenoids depending on mass-to-solvent mass ratios of SO and CLA.

Solid-to-Solvent Ratio	BC	LYC	Carotenoids **
		mg/100 g Dry Matter	
SO 1:10	3.97 ± 0.62	7.98 ± 0.97 *	11.95 ± 1.59 *
SO 1:20	3.89 ± 0.57	5.79 ± 0.09	9.68 ± 0.66
SO 1:30	3.25 ± 0.06	5.61 ± 0.21	8.86 ± 0.27
CLA 1:10	5.42 ± 0.18	10.7 ± 0.05 *	16.12 ± 0.63
CLA 1:20	5.88 ± 0.29	11.5 ± 0.05	17.38 ± 0.94
CLA 1:30	5.59 ± 0.12	11.0 ± 0.19	16.54 ± 0.91

* Value is significantly different compared to other solid-to-solvent ratios of the particular solvent (*p* < 0.05). ** calculated as the sum of BC and LYC. SO: sunflower oil; CLA: capric acid–lauric acid 2:1; BC: β-carotene; LYC: lycopene.

**Table 5:** MAE extraction conditions and energy consumption during MAE for SO.

Run	Time (Min)	Power (W)	Yields (mg/100 g of Dry TPW)		Energy Consumption (kWh)
			BC	LYC	
1	7	300	2.93 ± 0.21	3.92 ± 0.25	0.035
2	4	217	2.82 ± 0.24	3.57 ± 0.27	0.014
3	7	700	3.13 ± 0.06	4.10 ± 0.04	0.082
4	4	500	2.66 ± 0.17	2.94 ± 0.36	0.033
5	1	300	2.57 ± 0.23	2.95 ± 0.39	0.005
6	4	783	2.84 ± 0.07	3.29 ± 0.23	0.052
7	4	500	2.93 ± 0.00	3.14 ± 0.04	0.033
8	4	500	2.77 ± 0.29	3.02 ± 0.19	0.033
9	4	500	2.76 ± 0.45	3.14 ± 0.14	0.033
10	1	700	2.69 ± 0.09	2.98 ± 0.35	0.012
11	8	500	2.96 ± 0.43	3.28 ± 0.32	0.067
12	4	500	2.64 ± 0.29	2.75 ± 0.21	0.033
13	7	1100	2.98 ± 0.19	3.06 ± 0.16	0.128
14	4	1265	2.76 ± 0.17	2.72 ± 0.21	0.084
15	13	1600	2.44 ± 0.22	1.53 ± 0.30	0.347
16	1	1600	3.17 ± 0.18	2.88 ± 0.12	0.027
17	1	100	3.11 ± 0.09	2.58 ± 0.09	0.002

BC: β-carotene; LYC: lycopene; TPW: tomato processing waste.

**Table 6:** MAE extraction conditions and energy consumption during MAE for CLA.

Run	Time (Min)	Power (W)	Yields (mg/100 g of Dry TPW)		Energy Consumption (kWh)
			BC	LYC	
1	7	300	3.62 ± 0.04	4.84 ± 0.15	0.035
2	4	217	2.99 ± 0.12	3.73 ± 0.27	0.014
3	7	700	4.10 ± 0.28	5.08 ± 0.36	0.082
4	4	500	3.20 ± 0.39	3.34 ± 0.46	0.033
5	1	300	4.11 ± 0.26	4.07 ± 0.12	0.005
6	4	783	4.44 ± 0.09	4.96 ± 0.05	0.052
7	4	500	3.22 ± 0.60	3.38 ± 0.79	0.033
8	4	500	3.37 ± 0.81	3.45 ± 0.75	0.033
9	4	500	3.54 ± 0.79	3.43 ± 0.85	0.033
10	1	700	3.58 ± 0.05	3.75 ± 0.05	0.012
11	8	500	4.19 ± 0.39	4.72 ± 0.44	0.067
12	4	500	3.97 ± 0.13	3.97 ± 0.13	0.033
13	7	1100	2.75 ± 0.27	3.57 ± 0.29	0.128
14	4	1265	3.28 ± 0.64	4.41 ± 0.68	0.084
15	13	1600	4.01 ± 0.13	6.57 ± 0.54	0.347
16	1	1600	3.05 ± 0.12	3.20 ± 0.11	0.027
17	1	100	2.61 ± 0.08	2.54 ± 0.11	0.002
18	20	1600	4.22 ± 0.35	7.58 ± 0.88	0.533
19	25	1800	4.85 ± 0.37	8.69 ± 1.48	0.750
20	13	1800	5.29 ± 0.84	9.80 ± 1.77	0.390
21	5	1800	3.20 ± 0.17	3.13 ± 0.32	0.150
22	13	1800	4.40 ± 0.56	7.36 ± 1.16	0.390
23	25	1800	4.30 ± 0.92	6.59 ± 2.15	0.750

BC: β-carotene; LYC: lycopene; TPW: tomato processing waste.

**Table 7:** Overview of environmental impacts of seven extraction methods of tomato pomace.

Environmental Potential	Unit	SO			CLA			HA
		CSE	MAE	UAE	CSE	MAE	UAE	CSE
Global warming	kg CO_2eq_	11.8399	11.6873	11.8458	11.8369	11.8619	11.8458	11.8222
Ozone depletion	kg CFC11_eq_	2.89 × 10^−7^	2.87 × 10^−7^	2.91 × 10^−7^	2.91 × 10^−7^	2.91 × 10^−7^	2.91 × 10^−7^	2.93 × 10^−7^
Terrestrial acidification	kg SO_2eq_	0.1336	0.1321	0.1339	0.1338	0.1341	0.1339	0.1336
Freshwater eutrophication	kg P_eq_	0.0191	0.0189	0.0192	0.0191	0.0192	0.0192	0.0191
Marine eutrophication	kg N_eq_	0.0145	0.0142	0.0146	0.0146	0.0144	0.0146	0.0141
Overall toxicity	kg 1.4-DCB	13.117	13.002	13.199	13.189	13.198	13.199	13.115
Water depletion	m^3^	0.0667	0.0648	0.0738	0.0737	0.0656	0.0738	0.0572
Land use	m^2^a crop_eq_	0.8739	0.6556	0.7020	0.7015	0.6649	0.7020	0.6241
Fossil resource depletion	kg oil_eq_	3.1219	3.0864	3.1268	3.1243	3.1328	3.1268	3.1361

SO: sunflower oil; UAE SO: CLA: capric acid–lauric acid 2:1; hexane–acetone 1:1; CSE: conventional solvent extraction; UAE; ultrasound-assisted extraction; MAE: microwave -assisted extraction.

## Data Availability

The raw data supporting the conclusions of this article will be made available by the authors upon request.

## Abbreviations

The following abbreviations are used in this manuscript:

MAE	Microwave-assisted extraction
TPW	Tomato processing waste
UAE	Ultrasound-assisted extraction
PEF	Pulsed electric field
PLE	Pressurized liquid extraction
LCA	Life-cycle assessment
SO	Sunflower oil
CLA	Capric acid and lauric acid
CSE	Conventional solvent extraction
DES	Deep eutectic solvent
GRAS	Generally recognized as safe
SCF	Short-chain fatty acid
TC	Total carotenoids
SFE	Supercritical fluid extraction
HPAE	High-pressure-assisted extraction
OO	Olive oil
MT	Menthol and thymol
MLA	Menthol and lactic acid
FT-IR	Fourier-transform infrared spectroscopy
DSC	Differential scanning calorimetry
TGA	Thermogravimetric analysis
HA	Hexane and acetone
LYC	Lycopene
BC	β-carotene
RP-HPLC	Reversed-phase high-performance liquid chromatography
FU	Functional units
GWP	Global Warming Potential
ODP	Ozone Depletion Potential
TAC	Terrestrial Acidification
FEP	Freshwater Eutrophication
MEP	Marine Eutrophication
OTX	Overall Toxicity
HTX	Human Toxicity
FTX	Freshwater Ecotoxicity
MTX	Marine Ecotoxicity
TTX	Terrestrial Ecotoxicity
WDP	Water Depletion
FDP	Fossil Fuel Depletion
ALO	Agricultural Land Occupation
CA	Capric acid
DW	Dry weight
DE	Dry extract
VM	Vibrational micro-milling
